# Inefficient Admissions for Abdominal Pain Under an Acute General Surgical Unit

**DOI:** 10.1007/s00268-023-07096-0

**Published:** 2023-06-23

**Authors:** Yash Panwar, Sam Shan, Lily Owens, Chiu Kang, Russell Hodgson

**Affiliations:** 1 Division of Surgery, Northern Health, 185 Cooper St, Epping, VIC 3076 Australia; 2 Department of Surgery, University of Melbourne, Epping, Australia

## Abstract

**Background:**

The acute general surgical unit (AGSU) model has become a standard of efficient acute surgical care. Whilst the AGSU has been compared to the traditional surgical model, there is a lack of research auditing referrals and admissions. This study evaluates abdominal pain referrals to AGSU and the necessity of admission.

**Methods:**

A retrospective cohort study of adult abdominal pain admissions was conducted over a two-year period at a single centre in metropolitan Victoria, Australia. The data were extracted from electronic medical records and key endpoints of data included the diagnosis, length of stay, investigations and subjective pain outcomes.

**Results:**

A total of 1587 patients met the study criteria of which 1116 (70.3%) had a non-surgical diagnosis with the majority having non-specific abdominal pain. The non-surgical patients had a lower median length of stay (25.3 h) compared to surgical patients (44.2 h, *p* < 0.001). They were less likely to have an abnormal haemoglobin (*p* = 0.004), elevated white cell count (*p* = 0.02) or elevated C-reactive protein > 50 mg/L (*p* < 0.001). On multivariable analysis, surgical patients had higher odds of having a CRP > 50 mg/L (*p* = 0.024) and a positive imaging result (*p* < 0.001). The patient’s pain control also correlated with length of stay.

**Conclusion:**

A large population of patients with non-specific abdominal pain are admitted to AGSU. These patients do not require surgery and have a short length of stay. Incorporating a negative CRP result and negative imaging result may be utilised in conjunction with optimised analgesia to help avoid these unnecessary admissions, thereby improving AGSU efficiency and workload.

## Introduction

The acute general surgical unit (AGSU) is a fast-paced surgical unit dedicated and designed for efficient acute general surgical care. It receives the hospital’s general surgical and trauma admissions, providing a diagnostic and therapeutic plan for these patients. Whilst this model has become the standard of surgical practice, there is limited evidence on improving its efficiency [1, 2].

The AGSU model was developed after observing an increase in key performance indicators including length of stay (LOS), time to surgical review in emergency department (ED), time to operating theatre and outcomes in general surgery, as well as an objection to the conflicting demands of balancing emergency and elective surgery with increased after hours operating that would potentially result in suboptimal care [3–5]. Initially developed in 2005 in the Prince of Wales Hospital in Sydney, then adopted by the Nepean hospital in 2006, it is now utilised across Australia and overseas [3, 4, 6, 7]. The AGSU has been shown to be superior to the traditional ‘on-call’ model of surgical admissions with reduced LOS, reduced time to surgery and reduced after hours operating following the implementation of an AGSU [3, 5, 6]. These and other studies have been conducted to compare the AGSU with the traditional model based on the outcomes from common surgical pathology and procedures such as appendicectomy and cholecystectomy [7].

Whilst the AGSU has been compared to the traditional model for the improvement of care in confirmed surgical pathologies, there is a paucity of research evaluating the referrals to AGSU and the management of patients without a defined surgical pathology. Non-essential referrals and admissions increase the workload for the surgical team, reducing efficiency and depleting financial resources [8, 9].

This retrospective review of a single centre AGSU assesses non-specific abdominal pain admissions with the aim of identifying factors in these patients that may allow identification of patients that can be treated more efficiently in other departments or as an outpatient. The results of this will provide greater clarity in designing models for efficient AGSU referrals and admissions, potentially resulting in improved patient flow, care and health economics.

## Methods

A retrospective cohort study was conducted of all patients who were admitted under the acute general surgical unit (AGSU) at Northern Health (Epping, Victoria, Australia) between March 31st, 2017 and March 31st, 2019 and admitted with the diagnosis of ‘Abdominal Pain’ as per the ICD10 code.

The population group was adults aged 18 years of age and older who were admitted from the emergency department. Patients less than 18 years of age and trauma admissions were excluded. The study data were collected from electronic medical records (EMR) and the inpatient administration system (iPM). The primary outcome assessed was the discharge and final diagnosis with the secondary outcome being the length of stay.

A non-surgical diagnosis was defined as those patients who did not undergo a procedure or have a surgical diagnosis at discharge or follow-up. The ‘surgical’ group of patients was defined as those patients who underwent a surgical procedure regardless of outcome, or non-operative patients who had a surgical diagnosis during the admission or follow-up based on clinical information and investigations.

Data that were collected included demographical data, investigations performed, surgical interventions, discharge diagnosis, follow-up disposition and final diagnosis. Time parameters including emergency and total hospital length of stay (LOS) were also recorded. The results of pathology testing including haemoglobin (Hb), white cell count (WCC) and C-reactive protein (CRP) were classified and analysed as the proportion abnormal. This was based on the values in relation to the standardised laboratory ranges in which normal WCC ranged from 4 to 11 × 10^6^ mmol/L. The normal haemoglobin values were 115–165 and 130–180 × 10^6^ mmol/L in females and males, respectively. Abnormal CRP values were classified as proportion greater than or equal to 10 and 50 mg/L, respectively.

There was no standardised pain evaluation data available; however, medical and nursing documentations were reviewed for patient’s level of pain prior to discharge. The subjective descriptions were classified as resolved, improved with analgesia, controlled, ongoing or not recorded.

Statistical analysis was performed with SPSS (SPSS Inc. Version 27, Chicago, Illinois, USA). Median and interquartile range values were selected for descriptive analysis of nonparametric continuous data. Statistical analysis of continuous data was performed with the independent samples *T*-test for parametric data and the Mann–Whitney U-Test for nonparametric data with significance set at a 5% alpha level. Discrete data were statistically analysed with the Chi-square test. Multivariable analysis was performed through binomial logistic regression.

Ethical approval was obtained from the Northern Health Research and Governance Office (ALR 53.2019).

## Results

**Fig. 1:**
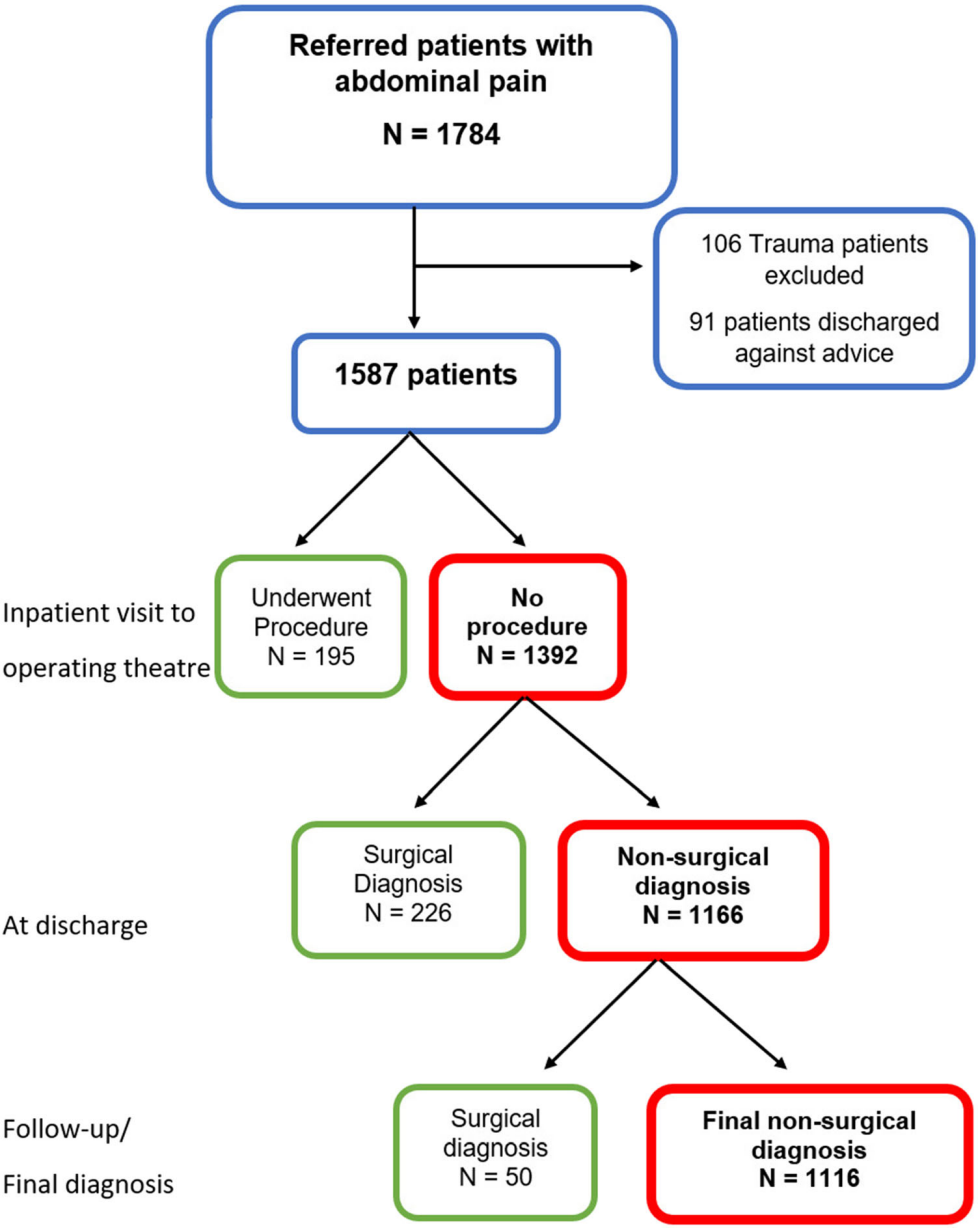
Flowchart of AGSU abdominal pain admissions

In the study period, there were 1784 admission episodes for abdominal pain. Figure 1 is a flowchart of the patients from admission through to outcome. There were 106 trauma admissions which were excluded from further analysis. There were a further 91 patients who had discharged against medical advice and were excluded from analysis.

The primary outcome was the proportion of patients who had a non-surgical cause for abdominal pain, and these were compared with the surgical patients (Table 1). Of the non-surgical causes of abdominal pain, non-specific abdominal pain was the most prevalent diagnosis overall, contributing to 71.8% of those diagnoses.

**Table 1:** Comparison of surgical versus non-surgical groups

	Surgical	Non-surgical	*p* value
n (%)	471 (29.7%)	1116 (70.3%)	
Median age (IQR)	44 [29–64]	40 [27–57]	*p* = 0.001
*Gender, n (%)*			*p* = 0.024
Male	180 (38.2%)	361 (32.3%)	
Female	291 (61.8%)	755 (67.7%)	
Hb (% low)	12.3%	7.7%	*p* = 0.004
WCC (% abnormal)	35.9%	29.7%	*p* = 0.02
*CRP*			
Elevated > 10	58%	46.3%	*p* < 0.001
Elevated > 50	20.5%	11.5%	*p* < 0.001
Imaging performed	88.3%	91.8%	*p* = 0.03
CT	61.8%	57%	*p* = 0.08
US	41%	47.7%	*p* = 0.01
CT and US	21%	18.9%	*p* = 0.33
Other imaging	6.6%	6.1%	*p* = 0.71
Confirmed diagnosis on imaging	31.6%	4%	*p* < 0.001
*Median LOS (IQR)*			
Hours	44.2 [23.5–85]	25.3 [17.3–44.4]	*p* < 0.001
Days	1.8 [0.98–3.5]	1.06 [0.72–1.85]	*p* < 0.001
ED LOS	8.8 [5.1–13]	7.9 [5–12]	*p* = 0.11
*Follow-up*			
GP/non-surgical	20.7%	54.1%	*p* < 0.001
Surgical outpatients	74.4%	41.2%	*p* < 0.001
Readmissions	4.9%	4.7%	*p* = 0.85

*IQR* interquartile range, *Hb* haemoglobin, *WCC* white cell count, *CRP* C-reactive protein, *CT* computed tomography, *US* ultrasound, *LOS* length of stay, *ED* emergency department, *GP* general practitioner

Blood test investigations were performed on all but one patient in the study. In the surgical group, there was a significantly higher number of patients with a haemoglobin below normal (12.3 vs 7.7%, *p* = 0.004), a WCC above normal (35.9 vs 29.7%, *p* = 0.02), and median CRP above 10 mg/L (58 vs 46.3%, *p* = 0 < 0.001) and above 50 mg/L (20.5 vs 11.5%, *p* < 0.001). Imaging investigations were conducted in 90% of the patients with CT scanning used more prevalently in surgical patients (61.8 vs 57%, *p* < 0.08), but conversely, ultrasound scanning was used less frequently (41 vs 47.7%, *p* = 0.01). LOS was a key outcome assessed and demonstrated a significantly longer LOS for surgical patients (median LOS 44.3 h vs 25.3 h, *p* < 0.001).

Significant factors from univariate analysis were selected for multivariable analysis (Table 2). The surgical group patients had significantly higher odds of being male, having an elevated CRP > 50 and having abnormal imaging results.

**Table 2:** Multivariable analysis of variables for having a surgical diagnosis

	Odds ratio	95% CI	*p* value
Age	1.006	0.99–1.014	*p* = 0.18
Gender (male)	1.41	1.02–1.95	*p* = 0.04
Low Hb	1.35	0.83–2.2	*p* = 0.24
Abnormal WCC	1.25	0.9–1.73	*p* = 0.17
CRP > 50	1.6	1.07–2.4	*p* = 0.024
Imaging performed	0.48	0.1–2.3	*p* = 0.48
Positive imaging diagnosis	14.1	9.3–21.3	*p* < 0.001

*Hb* haemoglobin, *WCC* white cell count, *CRP* C-reactive protein

**Fig. 2:**
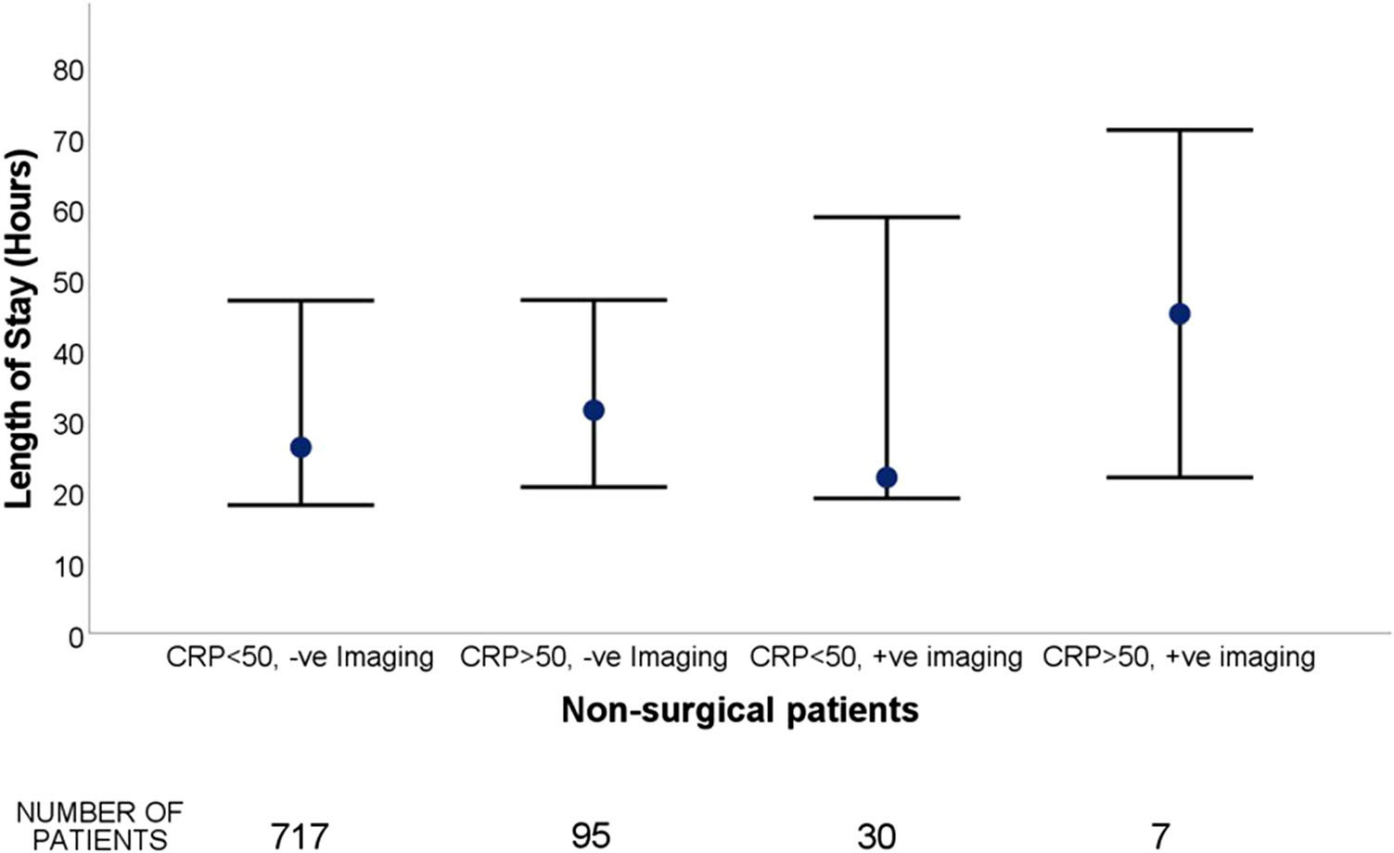
Graph of length of stay (median and IQR) for CRP and imaging diagnosis in non-surgical patients

Given the demonstrated utility of CRP and imaging, these were examined in more detail in the non-surgical group (Fig. 2). Of the 849 non-surgical patients that had CRP and imaging investigations performed. 84.5% had a CRP value below 50 mg/L with normal imaging. Not surprisingly, patients who had an elevated CRP > 50 mg/L and abnormal imaging had a longer median LOS (45 h) compared with other patients. Interestingly, patients with a CRP < 50 but with positive imaging had the shortest median LOS of 22 h albeit with a large IQR [IQR 18.5–66]. Non-surgical patients with a normal CRP and abnormal imaging had the second shortest median LOS of 26.2 h with the narrowest interquartile range [18–46.9]. On multivariable analysis of factors associated with a length of stay greater than 24 h, there were higher odds of having a CRP > 50 and having abnormal imaging results (Table 3).

**Table 3:** Multivariable analysis of variables for length of stay > 24 h

	Odds ratio	95% CI	*p* value
Age	1.001	0.99–1.01	*p* = 0.85
Gender (male)	0.82	0.64–1.05	*p* = 0.12
Low Hb	1.145	0.76–1.73	*p* = 0.52
Abnormal WCC	0.95	0.74–1.22	*p* = 0.7
CRP > 50	1.56	1.09–2.23	*p* −0.02
Imaging performed	1.27	0.5–3.2	*p* = 0.6
Positive imaging diagnosis	1.55	1.06–2.26	*p* = 0.02

*Hb* haemoglobin, *WCC* white cell count, *CRP* C-reactive protein

**Fig. 3:**
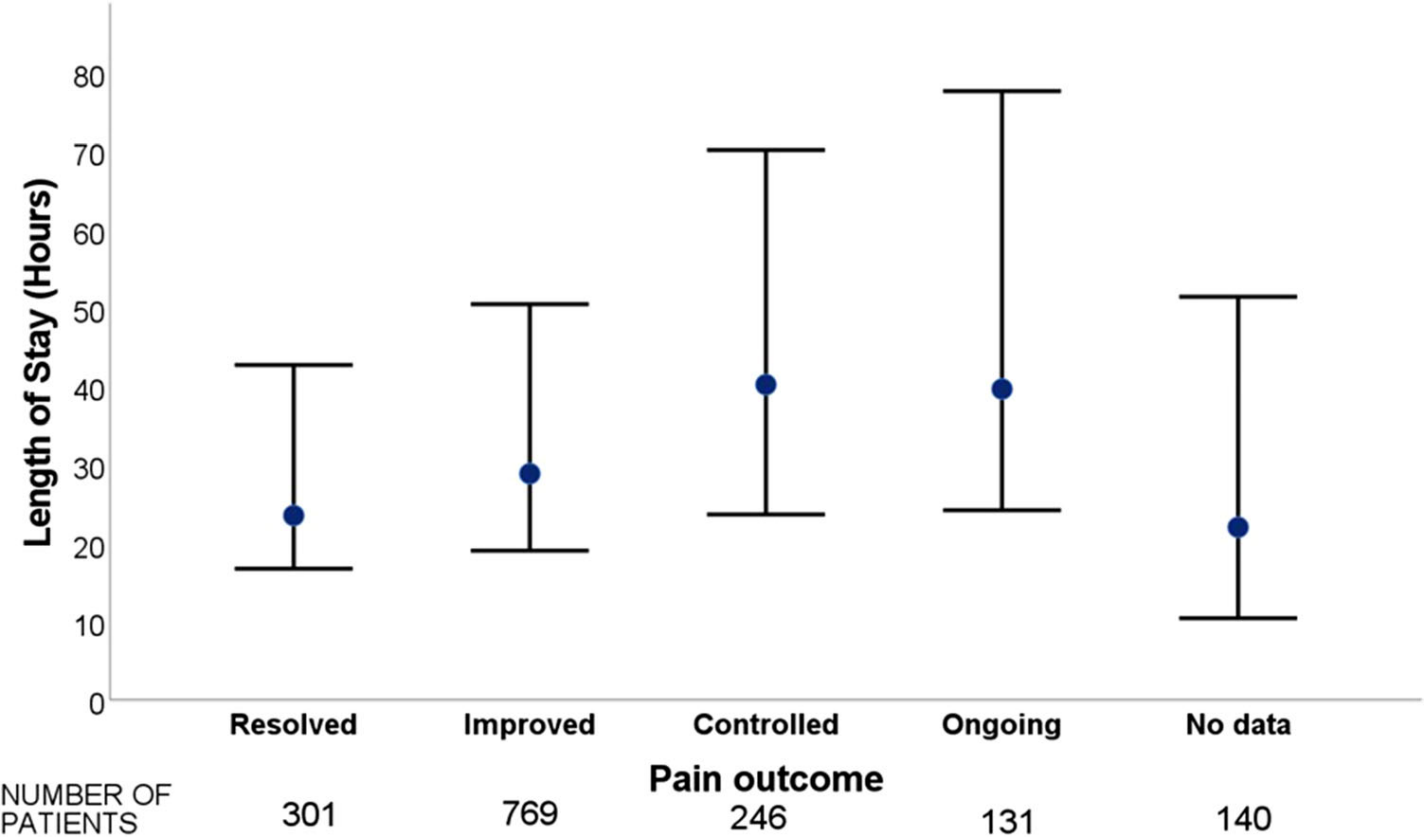
Graph of length of stay (median and IQR) for level of pain

Resolution of pain was examined as a further factor that may prevent or delay discharge and this data were available in 1447 patients. Resolution of pain was documented in 19% of patients whilst 48.5% had an improvement. Pain was controlled in 15.5% of patients and it was ongoing in 8.3%. There were no data available for the remaining 140 patients in the analysis. The LOS was compared across the pain outcome groups with the lowest median LOS (23.4 h) and shortest IQR [16.5–42.7] seen in the group with resolved pain (Fig. 3). Worse control of pain was correlated with a longer length of stay with the group that had ongoing pain having a higher median LOS (39.5 h).

To further examine the fifty patients for whom a surgical diagnosis was made following discharge, these patients were assessed for outpatient investigations and procedures (Table 4). These patients were not dissimilar in admission parameters from the entire cohort and the majority of these patients were diagnosed based on outpatient radiology and endoscopy/colonoscopy with diagnoses of GERD/gastritis/ulcer disease and biliary/gallstone pathology most common. Pain levels were largely reported as improved or resolved and less than 50% of these patients were discharged on an opiate based analgesic plan.

**Table 4:** Outcomes for patients diagnosed after discharge

n (%)	50 (100%)
Median age (IQR)	51 [39–67]
*Gender, n (%)*	
Male	18 (36%)
Female	32 (64%)
Hb (% low)	4 (8%)
WCC (% abnormal)	24 (48%)
*CRP*	
Elevated > 10	29 (58%)
Elevated > 50	6 (12%)
*Median LOS (IQR)*	
Hours	29 [20–70]
ED LOS	9 [6.1–15.8]
Discharged on opiates	23 (46%)
Oxycodone	20 (40%)
Oxycodone (slow release)	5 (10%)
Codeine	1 (2%)
*Reported pain at discharge*	
Resolved	20 (40%)
Improved	16 (32%)
Controlled	10 (20%)
Ongoing	3 (6%)
Not reported	1 (2%)
*Follow-up tests*	
Radiological scan	9 (18%)
Endoscopy/colonoscopy	36 (72%)
Required surgery	7 (14%)
*Surgical diagnosis*	
GERD/gastritis/ulcer disease	32 (64%)
Biliary/gallstone disease	7 (14%)
Hernia	3 (6%)
Malignancy	1 (2%)
Other	7 (14%)

*IQR* interquartile range, *Hb* haemoglobin, *WCC* white cell count, *CRP* C-reactive protein, *LOS* length of stay, *ED* emergency department, *GERD* gastro-esophageal reflux disease

## Discussion

This study demonstrates that many patients are admitted to an AGSU with a diagnosis of non-specific abdominal pain, consuming considerable resources. The non-surgical group of patients was more likely to have normal results for haemoglobin, WCC, CRP as well as negative imaging to investigate their pain and exclude surgical diagnoses. The admitted non-surgical patients had a shorter length of stay than surgical patients (*p* < 0.001) and the majority were able to have outpatient follow-up. As the admissions are short, it prompts the question as to whether these patients even require admission in the first place. In the literature, admission of non-specific abdominal pain patients has been shown to decrease acute surgical theatre efficiency [10, 11]. The disposition for those non-specific abdominal patients in the literature similarly includes referral to other specialties, diversion to outpatient management or patients requiring no specialist input [10]. The variables of normal haemoglobin, white cell count, CRP and negative imaging investigations may be utilised to avoid an admission for this population.

The recent increase in admissions for non-specific abdominal pain has coincided with the introduction of the ED four-hour rule (Australia and UK) and six-hour rule (New Zealand) [12, 13]. Whilst this study has not investigated changes in AGSU admission over time, this may be one explanation for the relatively higher proportion of admissions with non-specific abdominal pain. Increasing efficiency in ED, however, may not produce economic advantage. The cost benefits of AGSU are due to a reduced hospital length of stay as opposed to a reduced time in ED [7]. Admitting a larger number of patients with self-resolving symptoms can also produce artificially lower lengths of stay and morbidity/mortality rates [13, 14]. Ultimately, whilst AGSU creates an easily accessible and efficient pathway for surgical pathology to get rapid access to surgical management, the dedication of resources on patients not requiring surgical care will eventually result in slower access in care for all patients. Addressing barriers to discharge for these patients could help prevent admission and minimise length of stay.

Pain control can be a significant component to early discharge for patients with non-specific abdominal pain. As emphasised by the findings in Fig. 3, length of stay was inversely correlated with pain control regardless of diagnosis. The majority of acute abdominal pain referrals in the outpatient setting are settled with analgesia, reassurance and appropriate follow-up [10]. A focus on effective analgesic regimes in the GP or emergency department setting may prevent AGSU admissions for ongoing abdominal pain without a surgical cause in addition to reducing hospital length of stay for all patients.

Between 40 and 75% of GP abdominal pain referrals have been found to be appropriate to discharge from ED or treat in the general practice setting, and suggest the possibility of defensive medicine with patients referred based on an unclear diagnosis as opposed to a clear surgical indication[10, 15]. This is also reflected in the data from this study, with those patients with a CRP < 50 and confirmed diagnosis on imaging having the shortest length of stay, suggesting a fear of discharging patients without a diagnosis. However, a limitation of this study in making these comparisons is that only patients who were admitted under AGSU were assessed, with an unknown number of non-specific abdominal pain patients being successfully treated by ED without AGSU admission. Evidently from this study’s data, most admitted patients are discharged from hospital without a clear diagnosis in any case. A dedicated pathway for these patients would help alleviate the fear of discharging a patient without a known pathology.

Alternatives to a surgical admission include utilising a clinical decision unit or follow-up in an ambulatory clinic. The UK has adopted an emergency surgery ambulatory care pathway for appropriate diagnoses such as NSAP with these ‘hot clinics’ offloading up to 30% of presentations [16, 17]. Utilising a clinical decision unit is also an alternative for undifferentiated abdominal pain that requires admission with the majority being discharged home [18]. Multiple other studies have outlined the utility of outpatient clinic follow-up, providing a serial review to improve diagnostic accuracy whilst being comparable to emergency department observation and without a negative impact on outcome [19, 20]. Outpatient follow-up can therefore provide a valuable buffer for patients who continue to receive effective clinical care. Establishing and assessing the effectiveness of this management strategy are one of the future aims for our health service.

Selecting appropriate patients for and utilising the outpatient services can reduce the burden on AGSU and provide enhanced efficiency as well as avoiding unnecessary admissions for patients. Whilst studies assessing combined radiology and pathology investigations are lacking, early routine CT for abdominal pain has been found to increase surgical diagnostic accuracy and confidence in addition to reduced 6-month mortality [21, 22]. The results of this study demonstrate that a combination of normal haemoglobin, WCC, CRP and a negative imaging result can be utilised together to help support a non-surgical diagnosis in favour of a surgical diagnosis. This can be employed as part of an algorithm to avoid unnecessary non-surgical admissions to AGSU whilst ensuring urgent surgical conditions is not overlooked. In our study, utilising this criteria, 585 patients would have been identified for avoiding an admission saving 1128 hospital bed days. The application of such an algorithm would result in significant financial benefit as well as resource liberation to ensure more efficient care of the surgical patients, reducing staff burden.

## Conclusion

A vast majority of non-specific abdominal pain patients admitted to the AGSU do not require an operation and have a short length of stay. Avoiding an admission in these patients may help improve AGSU efficiency, hospital patient flow and health economics.

Utilising an algorithm incorporating inflammatory markers and negative imaging results in addition to pain optimization may enable safe discharge of these patients without compromising care whilst enhancing the efficiency of the AGSU.
